# The complete mitochondrial genome of the gullet worm *Gongylonema pulchrum*: gene content, arrangement, composition and phylogenetic implications

**DOI:** 10.1186/s13071-015-0697-5

**Published:** 2015-02-15

**Authors:** Guo-Hua Liu, Yan-Qing Jia, Ya-Nan Wang, Guang-Hui Zhao, Xing-Quan Zhu

**Affiliations:** State Key Laboratory of Veterinary Etiological Biology, Key Laboratory of Veterinary Parasitology of Gansu Province, Lanzhou Veterinary Research Institute, Chinese Academy of Agricultural Sciences, Lanzhou, Gansu Province 730046 Peoples Republic of China; College of Veterinary Medicine, Northwest A&F University, Shaanxi Province Yangling, 712100 Peoples Republic of China

**Keywords:** Gongylonema pulchrum, Gongylonemiasis, Mitochondrial genome, Phylogenetic analyses

## Abstract

**Background:**

*Gongylonema pulchrum* (Nematoda: Gongylonematidae), a thread-like spirurid gullet worm, infects a range of mammalian definitive hosts, including cattle, pigs, equines, goats, primates and humans, and can cause gongylonemiasis.

**Methods:**

In the present study, the complete mitochondrial (mt) genome of *G. pulchrum* was obtained using Long-range PCR and subsequent primer walking. The phylogenetic position of *G. pulchrum* within the Spiruromorpha was established using Bayesian analyses of the protein-coding genes at the amino acid level.

**Results:**

The length of this AT-rich (75.94%) mt genome is 13,798 bp. It contains 12 protein-coding genes, two ribosomal RNA genes, 22 transfer RNA genes and one non-coding region. The gene arrangement is the same as those of *Thelazia callipaeda* (Thelaziidae) and *Setaria digitata* (Onchocercidae), but distinct from that of *Heliconema longissimum* (Physalopteridae). Phylogenetic analyses, based on the concatenated amino acid sequence data for all 12 protein-coding genes using Bayesian inference (BI) method, showed that *G. pulchrum* (Gongylonematidae) was more closely related to *Spirocerca lupi* (Spiruroidea) than other members of the infraorder Spiruromorpha.

**Conclusions:**

The present study represents the first mt genome sequence for the family Gongylonematidae, which provides the opportunity to develop novel genetic markers for studies of epidemiology, population genetics and systematics of this nematode of human and animal health significance.

**Electronic supplementary material:**

The online version of this article (doi:10.1186/s13071-015-0697-5) contains supplementary material, which is available to authorized users.

## Background

*Gongylonema pulchrum* Molin, 1857, known as the ‘gullet worm’ because of its location in the upper digestive system of its definitive hosts, has a worldwide distribution and is responsible for gongylonemiasis of humans, sometimes causing serious complaints, e.g., expectoration of blood, numbness of tongue, vomiting, pharyngitis and stomatitis [1]. *G. pulchrum* also infects domestic and wild animal hosts, including bears, camels, cattle, cervids, donkeys, equines, goats, non-human primates, pigs, and sheep, causing major economic losses [2]. Fortunately, gongylonemiasis can be treated effectively using anthelmintics, such as levamisole, mebendazole and ivermectin [3].

Human gongylonemiasis, a neglected parasitic disease, has been reported from many countries (e.g., Australia, Bulgaria, Ceylon, China, France, Germany, Hungary, Iran, Japan, Laos, Morocco, New Zealand, Soviet Union, Spain, Sri Lanka, Turkey and USA). Recently, human gongylonemiasis has frequently been reported from China [4], France [5,6] and USA [7]. Clearly, the increased frequency and more widespread occurrence of clinical cases of human gongylonemiasis mean that knowledge on enhanced diagnosis, treatment and control is needed. Although molecular markers in portion of mitochondrial (mt) DNA and the internal transcribed spacer (ITS) regions of nuclear ribosomal DNA (rDNA), have found utility for taxonomic and epidemiological studies of *G. pulchrum* [8], there is still a paucity of information on *G. pulchrum* in different hosts and countries around the world*.*

Due to its maternal inheritance, fast rate of evolutionary change, lack of recombination and relatively conserved genome structures [9], mt genomes have been widely used as genetic markers for population genetic structure and the study of phylogenetic relationships among nematodes and trematodes [10]. In particular, concatenated amino acid sequences, derived from the protein-coding genes, lend themselves for assessing systematic relationships of parasitic nematodes [11-19]. The objectives of the present study were to characterize the mt genome of *G. pulchrum*, the first representative of the family Gongylonematidae, and to assess the phylogenetic position of this zoonotic nematode in relation to other nematodes of the infraorder Spiruromorpha.

## Methods

### Ethics statement

The performance of this study was strictly according to the recommendations of the Guide for the Care and Use of Laboratory Animals of the Ministry of Health, China, and our protocol was reviewed and approved by the Research Ethics Committee of Northwest A&F University.

### Parasite collection

Adult specimens of *G. pulchrum* were collected from the oesophagus of a naturally infected goat in Shenmu, Shaanxi province, China, with no specific permits being required by the authority for the sample collection.

### Genomic DNA extraction

The gullet worms were washed extensively in physiological saline, identified morphologically to species according to existing keys and descriptions [20], fixed in ethanol and then stored at −20°C until use. The mid-body section of each worm was used for the isolation of total genomic DNA using proteinase K digestion and mini-column purification (TIANamp Genomic DNA Purification System, TIANGEN). The identity of the specimen was verified by sequencing regions of the ITS-1 and ITS-2 rDNA using an established method [8]; the two regions were 99.7% and 100% identical to previously published sequences for *G. pulchrum* from *Bos taurus* in Iran (GenBank accession nos. AB495392 and AB513721, respectively).

### Long-PCR and sequencing

**Figure 1:**
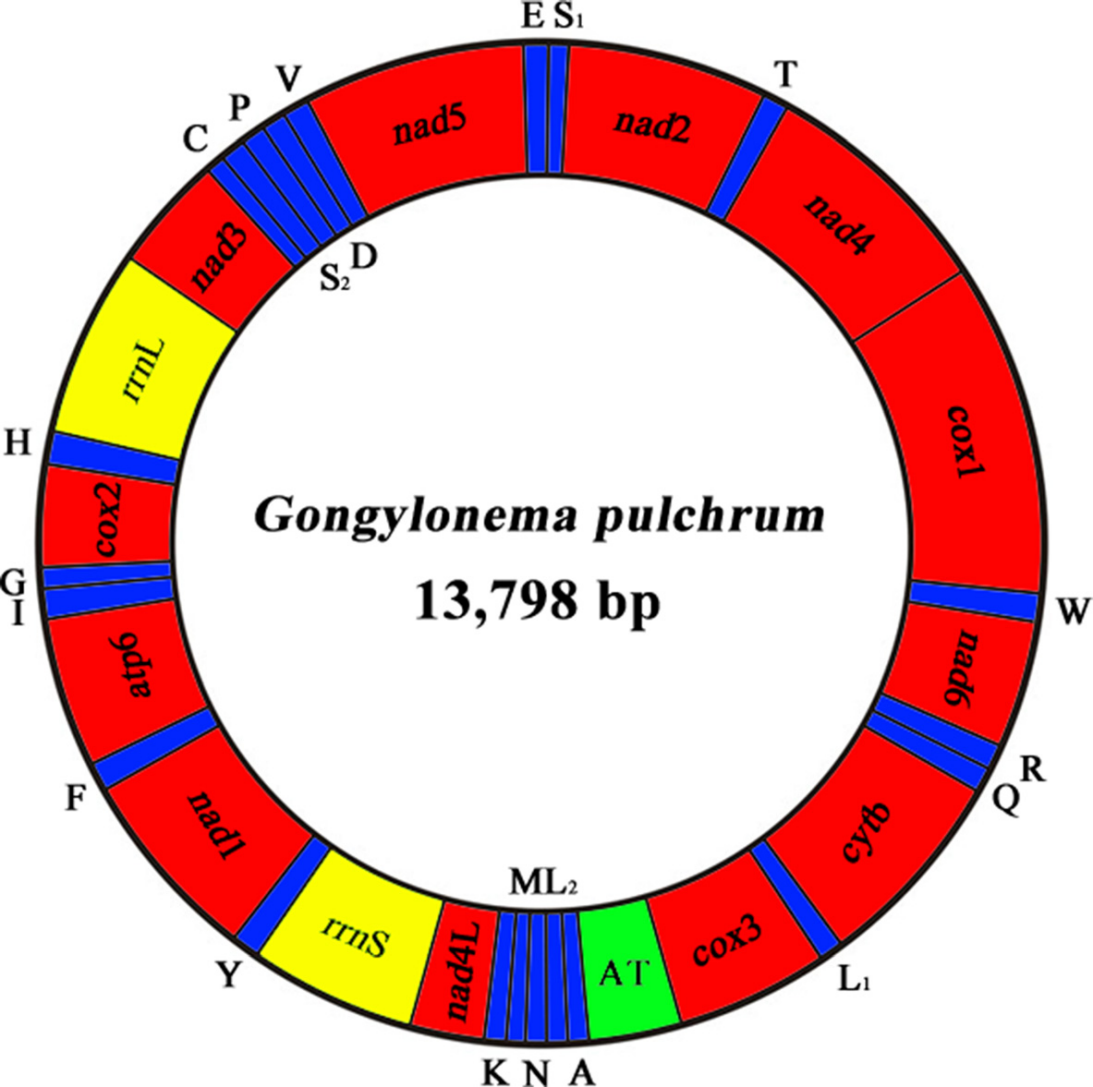
**Organization of the mitochondrial genome of** ***Gongylonema pulchrum*** **.** Scale is approximate. All genes have standard nomenclature except for the 22 tRNA genes, which are designated by the one-letter code for the corresponding amino acid, with numerals differentiating each of the two leucine- and serine-specifying tRNAs (L_1_ and L_2_ for codon families CUN and UUR, respectively; S_1_ and S_2_ for codon families UCN and AGN, respectively). All genes are transcribed in the clockwise direction. ‘AT’ indicates the non-coding region.

Using three pairs of specific primers (Table 1) designed according to relatively conserved regions within the *cox*1, *cox*2 and *cox*3 regions of nematodes within order Spirurata (Figure 1), three overlapping amplicons of the complete mt genome were amplified by Long-PCR [21]. PCRs were conducted in 25 μl reaction volumes containing 2 mM MgCl_2_, 0.2 mM each of dNTPs, 2.5 μl 10× Taq buffer, 2.5 μM of each primer and 0.5 μl LA *Taq* DNA polymerase (5 U/μl, TaKaRa). PCR cycling conditions were as follows: 92°C for 2 min (initial denaturation), then 92°C for 10 s (denaturation), 45°C for 30 s (annealing), and 60°C for 8 min (extension) for 9 cycles, followed by 92°C for 10 s, 45°C for 30 s (annealing), and 60°C for 9 min (extension) for 25 cycles, with a cycle elongation of 10 s for each cycle and a final extension at 60°C for 10 min. No-template and known-positive controls were included in each run. Amplicons were column-purified using TIANgel Midi Purification Kit (TIANGEN, Beijing, China). Following an electrophoretic analysis of quality, purified amplicons were sequenced using a primer walking strategy [21] with primers listed in Additional file 1 by Invitrogen Company (Shanghai, China).

**Table 1:** **Primers used to amplify the mitochondrial genome of** ***Gongylonema pulchrum*** **in three overlapping long PCR fragments**

**Name of primer**	**Sequence (5′ to 3′)**	**Extension positions**	**Product lengths**
cox3—cox2			
GPcox3u	TATGATATATCTATTGATTATTG	4162	3954 bp
GPcox2d	GCATCCATCTTAATAAAACACTTAGG		
cox2—cox1			
GPcox2u	GAGGTCGATAATCGTTGTATTATCCCTGTGG	8013	7155 bp
GPcox1d	AAGAATGAATAACATCCGAAGAAGT		
cox1 —cox3			
GPcox1u	TTTGGGGCTCCTGAGGTTTATA	770	3953 bp
GPcox3d	CAGAAATCTCTTCCATCACCTCGAT

### Sequence analyses

Sequences were assembled manually and aligned against the complete mt genome sequences of *Spirocerca lupi* [22] using the computer program MAFFT 7.122 [23] to identify gene boundaries. Each gene was translated into its amino acid sequence using the invertebrate mitochondrial genetic code in MEGA 5 [24]. The translation initiation and termination codons were identified to avoid gene overlap and to optimize the similarity between the gene lengths of closely related species of the infraorder Spiruromorpha. The program tRNAscan-SE [25] was used to find tRNA and infer their secondary structure, putative secondary structures of 19 tRNA genes were identified, and the remaining three tRNA genes (tRNA-Arg, tRNA-Ser ^AGN^ and tRNA-Ser ^UCN^) were inferred by recognizing potential secondary structures and anticodon sequences by eye [26]. Two rRNA genes were predicted by comparison with those of closely related nematodes of the infraorder Spiruromorpha [15,22].

### Phylogenetic analyses

Amino acid sequences inferred from published mt genomes representing 12 species of the infraorder Spiruromorpha (*Brugia malayi*: GenBank accession no. NC_004298; *Wuchereria bancrofti*: JN367461; *Chandlerella quiscali*: NC_014486; *Loa loa*: NC_016199; *Acanthocheilonema viteae*: NC_016197; *Onchocerca flexuosa*: NC_016172; *Onchocerca volvulus*: AF015193; *Dirofilaria immitis*: NC_005305; *Setaria digitata*: NC_014282; *S. lupi*: KC305876; *Thelazia callipaeda*: JX069968; *Heliconema longissimum*: NC_016127) were included in the present analysis, using *Toxascaris leonina* (NC_023504) as the outgroup [27]. 12 amino acid sequences were separately aligned using MAFFT 7.122 and then concatenated, with ambiguously aligned regions excluded using Gblocks 0.91b (doc) [28] with the default parameters using the options for a less stringent selection. Phylogenetic analyses were conducted using Bayesian inference (BI) method. The JTT+G+F model of amino acid evolution was selected as the most suitable model of evolution by ProtTest 2.4 [29] based on the Akaike information criterion (AIC). As the JTT model is not implemented in the current version of MrBayes, an alternative model, CpREV, was used in BI and four chains (three heated and one cold) were run simultaneously for the Monte Carlo Markov Chain. Two independent runs for 1,000,000 metropolis-coupled MCMC generations were used, sampling a tree every 100 generation in MrBayes 3.1.1 [30]. At the end of each run, the average standard deviation of split frequencies was less than 0.01. In addition, the potential scale reduction factor (~1) was examined to ensure that the convergence had been achieved. A 50% majority rule consensus tree was obtained from BI. Of 10,000 trees, the first 2,500 trees represented burn-in and the remaining trees were used to calculate Bayesian posterior probabilities (Bpp). Phylograms were drawn using the program FigTree v.1.4 [31].

## Results and discussion

The complete mt genomic sequence of *G. pulchrum* (GenBank accession no. KM264298) was 13,798 bp in size (Figure 1). It contains 12 protein-coding genes (*cox*1-3, *nad*1-6, *nad*4L, *atp*6 and *cyt*b), two ribosomal RNA (rRNA) genes, 22 transfer RNA (tRNA) genes and one non-coding (control or AT-rich) region, but lacks the *atp*8 gene (Table 2). The gene content and arrangement are the same as those of *T. callipaeda* (Thelaziidae) [15] and *S. digitata* (Onchocercidae) [32], but distinct from those of *D. medinensis* (Dracunculidae) and *H. longissimum* (Physalopteridae) [12]. All genes are transcribed in the same direction. In addition, the mt genome of *G. pulchrum* has 24 intergenic regions, ranging from 1 to 78 bp in length. The longest region (78 bp) is between tRNA-Pro and tRNA-Asp genes (Table 2). The nucleotide content of the entire mt genome sequence of *G. pulchrum* is biased toward A+T (75.94%), in accordance with mt genomes of other spirurid nematodes (Table 3). AT- and GC-skews of the whole mt genome were calculated for *G. pulchrum* and other spirurid nematodes studied to date (Table 4). The composition of the mt genome sequence of *G. pulchrum* was strongly skewed towards T (AT skew = −0.413), and G (GC skew was 0.448) (Table 3).

**Table 2:** **Mitochondrial genome organization of** ***Gongylonema pulchrum***

**Gene/Region**	**Positions**	**Size (bp)**	**Number of aa** ^**a**^	**Ini/Ter codons**	**In**
*cox*1	1-1653	1653	550	ATG/TAA	+1
tRNA-Trp (W)	1659-1717	59			+5
*nad*6	1744-2175	432	143	ATT/TAA	+26
tRNA-Arg (R)	2194-2245	52			+18
tRNA-Gln (Q)	2246-2299	54			0
*cyt*b	2323-3386	1064	354	ATT/TA	+23
tRNA-Leu^CUN^ (L_1_)	3387-3442	56			0
*cox*3	3449-4222	774	257	TTG/TAA	+6
Non-coding region	4223-4656	434			0
tRNA-Ala (A)	4657-4713	57			0
tRNA-Leu^UUR^ (L_2_)	4715-4768	54			+1
tRNA-Asn (N)	4770-4828	59			+1
tRNA-Met (M)	4839-4895	57			+10
tRNA-Lys (K)	4902-4959	58			+6
*nad*4L	4968-5195	228	75	TTG/TAA	+8
*rrn*S	5196-5876	681			0
tRNA-Tyr (Y)	5877-5933	57			0
*nad*1	5940-6804	865	288	TTG/T	+6
tRNA-Phe (F)	6807-6863	57			+2
*atp*6	6866-7442	577	192	GTT/T	+2
tRNA-Ile (I)	7443-7498	56			0
tRNA-Gly (G)	7506-7561	56			+7
*cox*2	7565-8255	691	230	ATG/T	+3
tRNA-His (H)	8256-8315	60			0
*rrn*L	8316-9280	965			0
*nad*3	9281-9620	340	113	GTG/T	0
tRNA-Cys (C)	9622-9676	55			+1
tRNA-Ser^UCN^ (S_2_)	9677-9730	54			0
tRNA-Pro (P)	9731-9784	54			0
tRNA-Asp (D)	9863-9917	55			+78
tRNA-Val (V)	9921-9973	53			+3
*nad*5	9979-11560	1582	527	TTG/T	+5
tRNA-Glu (E)	11562-11618	57			+1
tRNA-Ser^AGN^ (S_1_)	11620-11671	52			+1
*nad*2	11684-12513	830	276	ATG/TA	+12
tRNA-Thr (T)	12514-12568	55			0
*nad*4	12574-13797	1224	407	GTT/TAG	+5

^a^The inferred length of amino acid sequence of 12 protein-coding genes; Ini/Ter codons: initiation and termination codons; In: Intergenic nucleotides.

**Table 3:** **Comparison of A+T content (%) of gene and region of the mt genomes of spirurid nematodes sequenced to date (alphabetical order), including** ***Gongylonema pulchrum*** **(in bold)**

**Gene/region**	**AV**	**BM**	**CQ**	**DI**	**DM**	**GP**	**HL**	**LL**	**OF**	**OV**	**SD**	**SL**	**TC**	**WB**
*atp*6	75.21	75.09	80.14	71.88	72.40	**78.73**	77.89	76.46	73.71	72.99	74.23	74.87	74.23	76.63
*cox*1	67.36	68.98	70.28	67.88	68.21	**68.6**	71.69	69.48	69.70	67.03	69.10	66.97	67.88	67.70
*cox*2	66.81	68.96	73.25	69.15	68.25	**71.64**	74.71	71.53	68.10	69.24	69.38	68.51	67.38	70.57
*cox*3	71.54	72.69	76.92	71.79	71.54	**73.0**	75.93	76.20	72.18	71.79	72.56	71.39	72.41	74.33
*cytb*	72.32	73.97	76.13	72.25	72.14	**74.72**	79.30	75.35	73.65	72.11	72.34	72.85	73.68	72.70
*nad*1	73.43	73.55	75.85	72.94	72.29	**72.95**	75.69	72.85	71.60	69.78	72.78	72.50	73.22	72.52
*nad*2	74.68	77.61	82.39	74.39	76.93	**77.86**	82.92	77.26	75.56	74.30	76.49	70.91	77.35	75.71
*nad*3	79.82	79.35	81.71	77.15	75.89	**82.94**	83.18	79.82	76.56	76.11	77.06	80.65	80.24	84.27
*nad*4	73.98	76.31	78.05	74.55	72.32	**76.23**	80.36	75.75	74.05	73.15	76.91	74.47	75.59	73.88
*nad*4L	76.89	82.08	83.33	77.37	74.39	**77.63**	82.05	81.09	77.73	78.60	76.76	76.75	80.17	80.66
*nad*5	71.93	74.81	78.17	73.75	73.64	**74.97**	78.93	74.03	73.62	72.87	74.81	72.88	73.82	74.69
*nad*6	77.19	81.46	82.89	80.57	76.26	**81.02**	81.74	81.98	81.11	79.11	82.44	77.56	80.17	80.04
*rrn*S	75.48	76.04	76.85	75.84	73.59	**78.56**	80.50	76.56	75.84	74.71	74.55	76.09	75.68	75.30
*rrn*L	77.78	80.78	80.25	79.55	76.70	**81.14**	81.81	78.65	77.71	76.95	79.40	79.05	77.43	79.01
AT-loop	83.37	85.11	86.49	85.91	74.75	**81.11**	96.75	83.68	79.93	85.32	86.36	88.50	79.57	83.71
Entire	73.54	75.46	77.67	74.16	72.72	**76.0**	79.11	75.54	74.17	73.30	75.14	73.73	74.57	74.59

Nematodes: AV: *Acanthocheilonema viteae*, BM: *Brugia malayi*, CQ: *Chandlerella quiscali*, DI: *Dirofilaria immitis*, DM: *Dracunculus medinensis*, GP: *Gongylonema pulchrum*, HL: *Heliconema longissimum*, LL: *Loa loa*, OF: *Onchocerca flexuosa,* OV: *Onchocerca volvulus*, SD: *Setaria digitata*, SL: *Spirocerca lupi*, TC: *Thelazia callipaeda*, WB: *Wuchereria bancrofti*, Entire: entire mt genome.

**Table 4:** **Nucleotide composition of the mt genomes of spirurid nematodes, including that of** ***Gongylonema pulchrum (in bold)***

**Species**	**Nucleotide frequency (%)**				**Whole genome sequence**		
	**A**	**T**	**G**	**C**	**A+T%**	**AT skew**	**GC skew**
*Acanthocheilonema viteae*	19.56	53.98	19.26	7.20	73.54	−0.468	0.456
*Brugia malayi*	21.60	53.86	16.82	7.72	75.46	−0.428	0.371
*Chandlerella quiscali*	23.02	54.65	15.92	6.41	77.67	−0.407	0.426
*Dirofilaria immitis*	19.26	54.90	19.28	6.56	74.16	−0.481	0.492
*Dracunculus medinensis*	20.12	52.60	20.75	6.53	72.72	−0.447	0.521
***Gongylonema pulchrum***	**22.27**	**53.67**	**17.42**	**6.64**	**75.94**	−**0.413**	**0.448**
*Heliconema longissimum*	26.22	.89	14.14	6.75	79.11	−0.337	0.354
*Loa loa*	20.78	54.76	17.73	6.73	75.54	−0.450	0.450
*Onchocerca flexuosa*	20.30	53.88	18.60	7.23	74.17	−0.430	0.440
*Onchocerca volvulus*	19.26	54.04	19.84	6.86	73.30	−0.474	0.486
*Setaria digitata*	19.42	55.71	18.14	6.72	75.14	−0.483	0.459
*Spirocerca lupi*	21.9	51.83	19.4	6.87	73.73	−0.406	0.477
*Thelazia callipaeda*	22.39	52.18	18.42	7.01	74.57	−0.40	0.449
*Wuchereria bancrofti*	20.14	54.45	18.20	7.21	74.59	−0.460	0.433

The most common initiation codon for *G. pulchrum* is TGG, followed by ATG, ATT, GTT and GTG (4, 3, 2, 2, 1 genes, respectively; Table 2). The most frequent complete termination codon is TAA (4 genes); *nad*4 is terminated with the codon TAG. Of the remaining genes, *nad*1, *nad*3, *nad*5, *atp*6 and *cox*2 are terminated with the abbreviated stop codon T, whereas *cyt*b and *nad*2 are terminated with the abbreviated stop codon TA. This is consistent with the arrangement in the mt genomes of other nematodes [27,33-35].

Twenty-two tRNA genes were predicted from the mt genome of *G. pulchrum* and varied from 52 to 59 bp in length. Twenty of the 22 tRNA genes (excluding two tRNA-Ser) have a predicted secondary structure with a 3–5 bp DHU arm and a DHU loop of 7–9 bases, in which the variable TψC arm and loop are replaced by a “TV-replacement loop” of 8–10 bases. As seen in almost all other nematode mtDNAs [36], the tRNA-Ser gene of *G. pulchrum* mt genome is equipped with a TψC arm and loop but lacks the DHU arm and loop, consisting of a 6–8 bp TψC arm, TψC loop of 4–6 bases and a variable loop of 4 bases. The majority of nematode mtDNA sequences usually contain two non-coding regions with significant size difference [37], but there is only one non-coding region (AT-rich region) in the mt genome of *G. pulchrum* which is located between *cox*3 and tRNA-Ala (Figure 1 and Table 2), with 81.11% of A+T content (Table 3). Furthermore, this region is devoid of consecutive sequences of [A] and [T], and there are no AT dinucleotide repeat sequences; these repeat regions have been reported in the mt genome of *A. simplex s.l.* and *S. digitata* [32,34].

Identification and differentiation of *G. pulchrum* has traditionally been based on morphological features. However, these criteria are often insufficient for specific identification and differentiation, particularly at the larval and/or egg stages. Molecular tools, using genetic markers in mt *cox*1 and ITS-1 region of nuclear rDNA, have been used to support clinical diagnosis and to assist in undertaking molecular identification and epidemiological investigations of *G. pulchrum* [8]. Because sequence polymorphism (heterogeneity) in ITS rDNA sequences occurs within individual spirurid specimens [38], mt genome sequences appear to be better solutions for such studies. Additionally, the *cox*1 sequences of *G. pulchrum* were further divided into multiple haplotypes and two groups of haplotypes (i.e. those from a majority of sika deer, wild boars and Japanese macaques and those from cattle and zoo animals, were clearly differentiated) [8]. Nonetheless, the *cox*1 is a relatively conserved mt gene in nematodes [13,16,39], and, to date, there is no genetic information for *G. pulchrum* from other mt genes.

**Figure 2:**
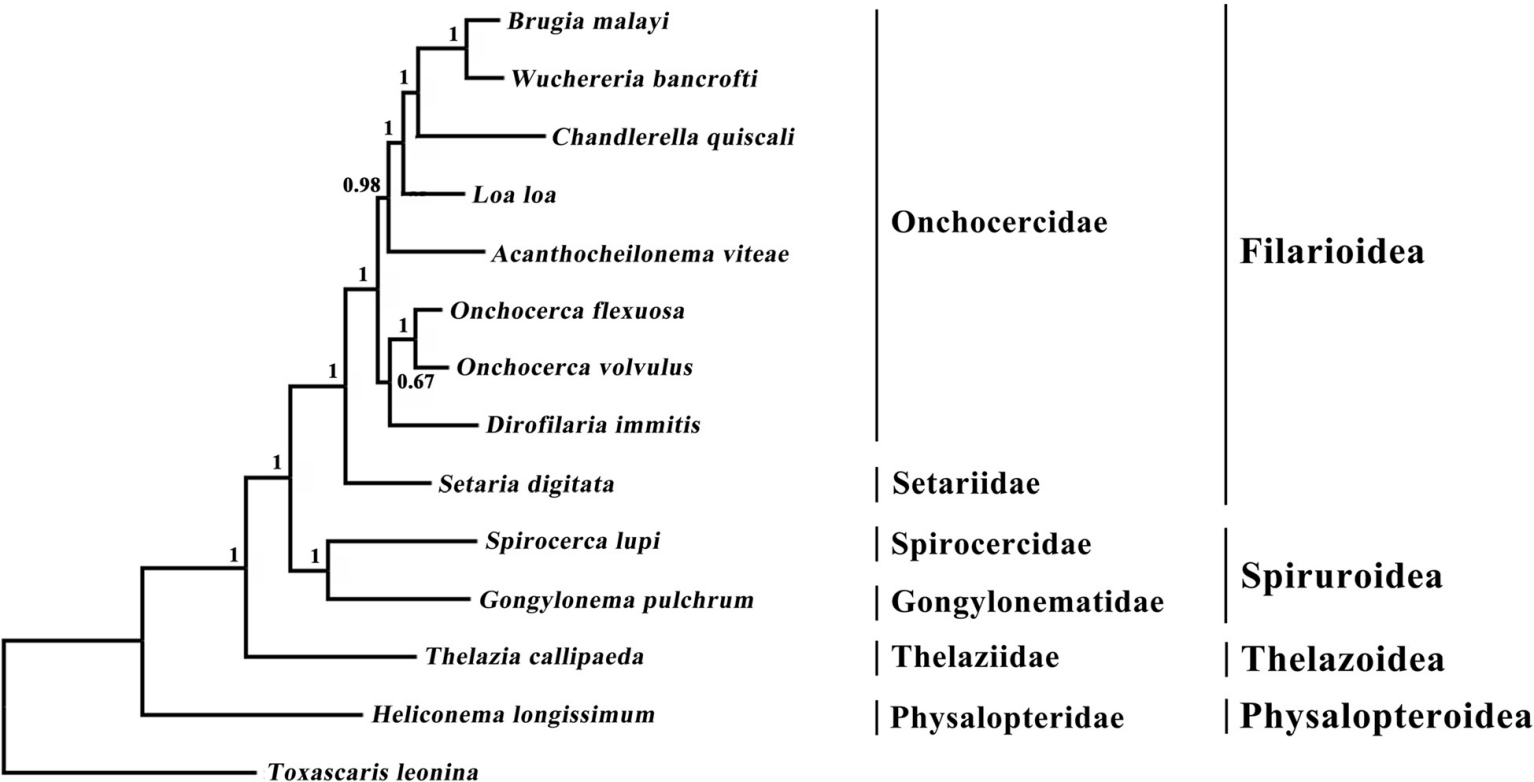
**Phylogenetic relationships of** ***Gongylonema pulchrum*** **with representative members of the infraorder Spiruromorpha based on mitochondrial sequence data.** The concatenated amino acid sequences of 12 protein-coding genes were analyzed using Bayesian inference (BI) using *Toxascaris leonina* as the outgroup. Bayesian posterior probability (Bpp) values are indicated.

Phylogenetic analyses of *G. pulchrum* with related nematodes of the infraorder Spiruromorpha were performed by BI based on concatenated mitochondrial amino acid sequences of 12 protein-coding genes (Figure 2). *Gongylonema pulchrum* (Gongylonematidae) formed the sister group to *Spirocerca lupi*. Together they formed the sister group to a clade composed of Setariidae and Onchocercidae. *Thelazia callipaeda* (Thelaziidae) took an early diverging position to the above mentioned taxa, whereas *Heliconema longissimum* (Physalopteridae) and *Toxascaris leonina* took an unresolved position at the root of the tree (Figure 2).

Many studies have indicated that the mtDNA sequence is a valuable genetic marker for phylogenetic studies [11,12,14,17]. The mt genome sequence of *G. pulchrum* could promote to reassess the systematic relationships within the spirurid nematodes using mt genomic datasets. Over the last decades, there have been considerable debate concerning the systematics of members of the spirurid nematodes (including superfamilies Filarioidea, Physalopteroidea, Spiruroidea and Thelazoidea) [40]. Some studies using nuclear small subunit (SSU) rDNA and mtDNA sequences have indicated that *S. lupi* (Spirocercidae) is the sister taxon of *T. callipaeda* (Thelaziidae), suggesting that *S. lupi* belongs to the superfamily Thelazioidea [41,42], but this finding was contradicted by other studies that used the same markers [43,44]. The results of the present study support that the Spirocercidae (represented by *S. lupi*) was more closely related to the family Gongylonematidae (represented by *G. pulchrum*) than to other families within Spiruromorpha, indicating that both families Spirocercidae and Gongylonematidae belong to superfamily Spiruroidea, consistent with conclusions of a previous study [40]. Given this utility of mt genomic datasets, further work should include sequencing of mt genomes of other spirurid nematodes in order to reconstruct the phylogenetic relationships of spirurid nematodes.

## Conclusions

The present study determined the complete mt genome sequence of *G. pulchrum*, and ascertained its phylogenetic position within the infraorder Spiruromorpha. The complete mt genome represents the first sequenced mt genome of any member of the family Gongylonematidae. It will provide an important resource for the design of novel primers for the study of epidemiology, population genetics and systematics of Gongylonematidae.

## Additional file

Additional file 1:
**Sequences of partial primer-walking primers used to amplify PCR fragments from** ***Gongylonema pulchrum.***
