# Supplementary material for: The complete mitochondrial genome of the gullet worm Gongylonema pulchrum: gene content, arrangement, composition and phylogenetic implications
Source: Parasit Vectors. 2015 Feb 15;8:100. doi: 10.1186/s13071-015-0697-5 (PMC4340675; doi:10.1186/s13071-015-0697-5)
Supplement: Additional file 1: — Sequences of partial primer-walking primers used to amplify PCR fragments from Gongylonema pulchrum. [file 13071_2015_697_MOESM1_ESM.doc]

**Additional_file_1.** Sequences of partial primer-walking primers used to amplify PCR fragments from *Gongylonema pulchrum*

| **Primer names** | **Sequence (5’ to 3’)** |
| --- | --- |
| GP*cox*3u | TATGATATATCTATTGATTATTG |
| GP*cox*2d | GCATCCATCTTAATAAAACACTTAGG |
| GP*cox*2u | GAGGTCGATAATCGTTGTATTATCCCTGTGG |
| W1F | CGTGTGCCAACATTATAACT |
| W1R | TGAAAGGTTGTGTTTTTGAC |
| W2F | AAATCCAATATAACCCAACA |
| W2R | AGCGGTTTATCTTGTATAAT |
| W3F | CAACACAACAAAATAAGGTA |
| W3R | CCTTTTGTGGTCTTTTAGTT |
| W4F | CCTTTAAACTCTAAAAATCC |
| NewR | GGACCACTGAGGAAAATCCCCCCGGGACCAG |
| GP*cox*1d | AAGAATGAATAACATCCGAAGAAGT |
| GP*cox*1u | TTTGGGGCTCCTGAGGTTTATA |
| GP*cox*3d | CAGAAATCTCTTCCATCACCTCGAT |
